# Kawasaki Disease, MIS-C and COVID-19

**DOI:** 10.3390/children10101587

**Published:** 2023-09-22

**Authors:** Ying-Hsien Huang, Ho-Chang Kuo

**Affiliations:** 1 Kawasaki Disease Center and Department of Pediatrics, Kaohsiung Chang Gung Memorial Hospital, #123 Da-Pei Road, Niao-Sung District, Kaohsiung 83301, Taiwan; yhhuang123@yahoo.com.tw; 2 College of Medicine, Chang Gung University, Taoyuan 33302, Taiwan; 3 Department of Respiratory Therapy, Kaohsiung Chang Gung Memorial Hospital, Kaohsiung 83301, Taiwan

Kawasaki disease (KD) is a form of systemic vasculitis characterized by inflammation of blood vessels throughout the body, and its exact cause remains unknown. Various medical specialists, including those in immunology, infectious diseases, rheumatology, and cardiology, have been involved in the study of KD, although a specific discipline has not been exclusively responsible. KD primarily affects children under the age of 5, and for the past two to three decades, intravenous immunoglobulin (IVIG) has shown a favorable treatment response. The clinical characteristics of KD include a fever lasting for more than 5 days, as well as at least four of the following five symptoms: diffuse mucosal inflammation of the tongue and lips (1—mouth), bilateral conjunctivitis (2—eyes), unilateral cervical lymphadenopathy with enlargement (3—fingers, checks, and lymph nodes), indurative angioedema over the limbs (4—limbs), and numerous skin rashes (5—this means a lot). These five major characteristic symptoms of KD may be easier to remember by using the 1–2–3–4–5 “Kuo Mnemonic”.

The most significant complication of KD is the development of coronary artery lesions (CALs), which can have long-term implications for the affected children and their families. Researchers have explored genetic and immunological factors as potential contributors to the pathogenesis of KD. Notably, there are over 7000 publications on KD available through the PubMed database, with a substantial focus on genes and immune responses. This Special Issue aims to gather articles from different regions and nations, specifically focusing on the genetics and immune responses in KD. By consolidating these findings, the goal is to gain a clearer understanding of the immunopathogenesis of KD and potentially shed light on its etiology. The pandemic caused by coronavirus disease 2019 (COVID-19) began, as the name suggests, in 2019 and has been ongoing since. The effect of COVID-19 on children is usually mild, but some are susceptible to a novel Kawasaki-like disease, also known as multisystem inflammatory syndrome in children (MIS-C). Kawasaki disease (KD) is different from MIS-C in terms of age, severity, clinical presentation, susceptibility, immune response, cause, and therapy. Coronary artery involvement, the BCG vaccination relationship, mucosa/skin manifestations, and treatment strategies have shown a great overlap between KD and MIS-C. This Special Issue focuses on articles outlining comparisons between KD and MIS-C.

A total of eight articles were accepted for publication on this research topic including four original research papers and four case reports.

Chen et al. [1] investigated the correlation between vascular endothelial growth factor (VEGF) and KD. They observed that KD patients who developed acute CALs exhibited higher median levels of VEGF compared to those without acute CALs, across the acute to convalescent phases of the disease. Notably, during the subacute phase, KD patients with acute CALs had significantly elevated VEGF levels in comparison to those without acute CALs. Importantly, their findings indicate that VEGF levels did not decrease following IVIG treatment but, instead, exhibited a significant increase in KD patients with acute CALs during the acute phase. These results suggest a potential association between VEGF and the development of CAL complications in KD patients.

Yang et al. [2] developed a novel predictor using the hemoglobin-for-age z-score (HbZ) and plasma hepcidin to differentiate KD from febrile children (FC). They developed an SVM model that achieved a sensitivity of 81.7% and a specificity of 91.3%, reaffirming its robust discriminatory ability. The implementation of this predictor in clinical practice can aid frontline physicians in promptly recognizing KD cases and facilitating early treatment initiation. The results indicate the importance of anemia in KD.

Campanello et al. [3] aimed to provide a comprehensive description of cardiovascular involvement, management strategies, and early outcomes in children diagnosed with multisystem inflammatory syndrome in children (MIS-C), with a specific focus on comparing cardiovascular manifestations in children. They found that cardiovascular involvement in MIS-C is characterized by distinct patterns in different age groups. Specifically, coronary artery anomalies were found to be more prevalent among the younger group, whereas myopericardial disease was more frequently observed in the older age group.

Huang et al. [4] aimed to develop a model utilizing the prognostic nutritional index (PNI) in conjunction with immunological factors to effectively differentiate between KD and other febrile illnesses. They reported that in comparison to children with febrile diseases, patients diagnosed with KD exhibited elevated levels of the C3 complement component and a decreased nutritional index regarding the PNI. Leveraging these factors, a nomogram was constructed, demonstrating its efficacy in accurately distinguishing KD from other febrile illnesses in children.

Maglione et al. [5] reported a rare case of lower motor neuron facial nerve palsy as a complication of KD. In this particular case, the patient’s diagnosis was made on the sixth day of the disease, and they also exhibited extensive coronary artery lesions. Furthermore, they also conducted an extensive literature review to enhance our understanding of the clinical features and treatment approaches employed in patients experiencing KD-associated facial nerve palsy.

Rodríguez-Fanjul et al. [6] analyzed the association between breastfeeding and MIS-C. Their findings do not provide substantial evidence to support the hypothesis that breastfeeding plays a preventative role in MIS-C.

Bossi et al. [7] presented the case of a 2-month-old male diagnosed with KD who experienced the complication of macrophage activation syndrome (MAS). Despite receiving prompt and intensive treatment including immunoglobulins, steroids, and aspirin, the patient developed multiple coronary artery aneurysms (CAAs). However, a therapeutic intervention involving a two-month course of anakinra resulted in the complete resolution of all the aneurysms. After a six-month period, the infant suffered a KD relapse, which was effectively managed through re-treatment with immunoglobulins, steroids, and aspirin.

Huang et al. [8] described the clinical presentation of a five-year-old boy who initially presented with symptoms of appendicitis. Despite undergoing an appendectomy, the persistence of fever prompted further evaluation, leading to a subsequent diagnosis of KD. IVIG therapy was administered, resulting in the resolution of symptoms and an uncomplicated recovery for the patient. Additionally, we conducted a comprehensive literature review, identifying a total of 13 reported cases with similar clinical presentations. Notably, the majority of these cases involved male patients, and the average age at presentation was higher than the typical age range associated with KD.

This Special Issue encompasses various studies that have contributed to a better understanding of the genetic and immune responses in KD and MIS-C. Additionally, through their work, the authors also shared their experiences of rare symptoms or signs of KD and have provided valuable insights for disease assessment and therapeutic decision-making in KD. Collectively, these findings contribute to our understanding of the disease process and offer valuable guidance in terms of disease status assessment and treatment selection for both KD and MIS-C.
